# Maternal Vitamin D Levels and Its Correlation With Low Birth Weight in Neonates: A Tertiary Care Hospital Experience in Saudi Arabia

**DOI:** 10.7759/cureus.14528

**Published:** 2021-04-16

**Authors:** Eyad Almidani, Abdullatif Barkoumi, Weam Elsaidawi, Saleh Al Aliyan, Abdulhakiem Kattan, Fahad Alhazzani, Mohammed bin Jabr, Abdulaziz Binmanee, Nada Alsahan, Saria Alazmeh

**Affiliations:** 1 Pediatrics, King Faisal Specialist Hospital and Research Centre, Riyadh, SAU; 2 Pediatrics, Alfaisal University College of Medicine, Riyadh, SAU; 3 Obstetrics and Gynaecology, King Faisal Specialist Hospital and Research Centre, Riyadh, SAU; 4 Medicine, Alfaisal University College of Medicine, Riyadh, SAU

**Keywords:** low birth weight, saudi arabia, vitamin d, birth weight, pediatrics & neonatology, public awareness of vitamin d, term neonates, preterm neonate, neonatal intensive care unit (nicu), maternal-child health

## Abstract

Introduction

A meta-analysis showed that 63.6% of the Saudi population have vitamin D deficiency, including many pregnant women. Studies showed that maternal vitamin D deficiency during pregnancy is a risk factor for low birth weight (LBW) in neonates. Neonatal LBW is a risk factor for multiple neonatal complications including respiratory distress syndrome, necrotizing enterocolitis, chronic renal disorders, seizures, and sepsis. Our objective in this study is to determine a correlation between low maternal vitamin D level and neonatal LBW in Saudi Arabia.

Methods

Neonates (n = 119) were divided based on their gestational age (GA) into full-term neonates (≥37 weeks) and preterm neonates (< 37 weeks) and based on birth weight into normal birth weight neonates (full-term = 2,500-3,500 g or preterm > 10th percentile) and LBW neonates (full-term < 2,500 g or preterm < 10th percentile). Vitamin D deficiency is defined as 25- hydroxyvitamin D level less than 50 nmol/L.

Results

Correlating neonatal birth weight with maternal vitamin D level during pregnancy was statistically insignificant for both full-term neonates and preterm neonates. In contrast, comparing the mean maternal vitamin D levels in each neonatal group showed that the mean were higher in mothers of neonates with normal birth weight.

Conclusion

Because 87.4% of mothers had low vitamin D levels during their pregnancy, correlation between maternal vitamin D level and LBW in neonates could not be found. However, mean maternal vitamin D levels were higher in mothers with normal birth weight neonates. Therefore, further detailed studies are required to establish local guidelines about the treatment of vitamin D deficiency during pregnancy.

## Introduction

Vitamin D is a fat-soluble vitamin produced in the body through the interaction of sunlight with 7-dehydrocholesterol (chemical precursor for vitamin D); the UV light breaks one of its chemical bonds to convert it into the active vitamin. Vitamin D is found in two forms: vitamin D3 (cholecalciferol) in humans and animals and vitamin D2 (ergocalciferol) in plants [1,2]. Vitamin D has a key role in bone mineralization by interacting with calcium and parathyroid hormone homeostasis [3].

Vitamin D concentration in the body depends on multiple factors such as by geographic location, latitude, skin pigmentation, and seasonal changes (in countries with a long winter season, vitamin D deficiency is very common especially among pregnant women). Also, nearly half of African-American pregnant women in the United States have vitamin D deficiency (vitamin D level less than 37.5 nmol/L), while it is less than 30% in Caucasian women. Concentration of 25 (OH)D in maternal circulation correlate with those found in the placental veins, which means that calcidiol easily passes through the placental barrier and the fetal vitamin D level depends mainly on the maternal vitamin D. In countries where the milk products are not supplemented with vitamin D, the dual-energy X-ray absorptiometry (DEXA) of the infants shows lower whole-body mineral value compared to infants in countries with supplemented milk products [4].

In a country like Saudi Arabia known for its sunny climate, you would expect most of the population to have sufficient levels of vitamin D, but a meta-analysis showed that 63.6% of the Saudi population have vitamin D deficiency, including many pregnant women. This could be related to multiple reasons such as inactivity of the community, diabetes mellitus, and obesity [5].

Studies recently showed that maternal vitamin D deficiency during pregnancy is a risk factor for low birth weight (LBW) in neonates [3,5]. In Saudi Arabia, 7.4% of the neonates are born with LBW (excluding stillbirths) [6], and their mortality rate is 40 times higher than normal birth weight neonates [3]. Neonatal LBW is a strong risk factor for multiple long-term and short-term neonatal complications including respiratory distress syndrome, necrotizing enterocolitis, chronic renal disorders, seizures, anemia, sepsis, and attention deficit hyperactivity disorder (ADHD) [7,8]. Also, it is a risk factor for multiple maternal disorders such as preeclampsia, insulin resistance and gestational diabetes mellitus, and higher risk of primary cesarean delivery [4].

Our objective in this study is to determine a correlation between low maternal vitamin D level and neonatal LBW in Saudi Arabia.

## Materials and methods

This is a retrospective cohort study including neonates born between November 2016 and October 2018 at King Faisal Specialist Hospital and Research Center (KFSH&RC), Riyadh, Saudi Arabia. Neonates were divided based on their gestational age into full-term neonates (≥ 37 weeks Gestational Age) and preterm neonates (<37 weeks’ gestational age). Also, neonates were divided based on their birth weight into normal birth weight neonates (full-term between 2,500-3,500 g or preterm > 10th percentile) and LBW neonates (full-term < 2,500 g or preterm < 10th percentile of the population) [6,9]. Then, maternal vitamin D level was correlated with neonatal birth weight and compared between all groups.

Vitamin D deficiency is defined as vitamin D level less than 50 nmol/L (20 ng/mL) of 25-hydroxyvitamin D, 25(OH)D, and insufficiency level is between 51 and 74 nmol/L (21-29 ng/mL) [5]. The acceptable maternal vitamin D (25-hydroxyvitamin FD 25(OH)D) level was considered the first measurement during pregnancy, and if not found, the last reading up to six months prior to pregnancy was considered valid.

A total of 147 pairs (mothers and neonates) were enrolled in this study, of which 18 were excluded because the maternal vitamin D measurement was not valid and 10 were excluded because of neonatal congenital anomalies such as trisomy 21, congenital pulmonary airways malformation, Dandy-Walker malformation, hypoplastic left heart syndrome, and homocystinuria. Furthermore, mothers aged below 18 and above 45 were excluded due to the low number of pregnant women at these ages. Accordingly, 119 neonates were included. The used data were obtained by collecting the medical record number of the included cases (mothers and neonates) through the hospital database (Integrated Clinical Information System [ICIS]). Data were presented as means ± standard deviation or as median and interquartile range for the continuous variables, where appropriate, after testing the normality. Categorical variables were presented as frequencies and valid percentages. Student’s t-test was used to assess the difference in means between full-term and preterm groups, and chi-square and Fisher’s exact tests were used where appropriate. The correlation between maternal Vitamin D and neonatal birth weight was made using Pearson’s correlation coefficient. A p-value of <0.05 was considered a threshold of significance. The analysis was conducted using SPSS Version 20 (IBM Corp., Armonk, NY, USA). This study was approved by the Institute Ethics Committee in KFSH&RC.

## Results

The neonates were divided based on the gestational age (Table 1); there were 64 full-term neonates (mean birth weight = 2.54 kg) and 55 preterm neonates (mean birth weight = 1.72 kg). The mothers’ average age was 30.7 years, and there were 104 mothers who had low vitamin D level at one point during pregnancy (87.4%), with an average vitamin D level of 44.97 nmol/L. For delivery method, 67 neonates were delivered by caesarean section and 52 through spontaneous vaginal delivery.

**Table 1:** Descriptive Information

Measure	Value	Frequency	Percentage
Gestational age	Full-term	64	53.8
	Preterm	55	46.2
Neonatal gender	Male	53	44.5
	Female	66	55.5
Maternal vitamin D level	Normal	15	12.6
	Low	104	87.4
Maternal diseases	Thyroid/parathyroid disorders	34	28.6
	Diabetes mellitus or gestational diabetes	24	20.2
	Hypertension	7	5.9
	Preeclampsia	2	1.7

**Figure 1:**
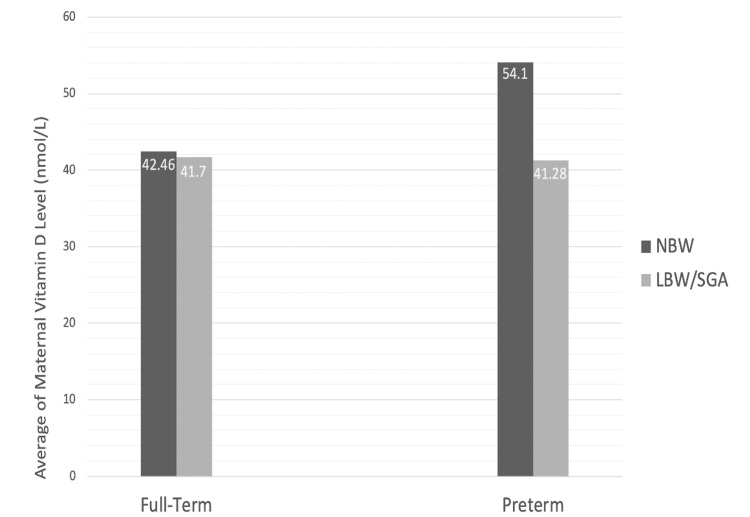
Mean Maternal Vitamin D Levels in Full-Term and Preterm Neonates LBW, low birth weight; NBW, neonatal birth weight; SGA, small for gestational age

After correlating the neonatal birth weight with the maternal vitamin D level during pregnancy, the result was statistically insignificant for both full-term neonates (p = 0.302) and preterm neonates (p = 0.736). In contrast, comparing the mean maternal vitamin D levels in each neonatal group (Figure 1) showed that full-term neonates with normal birth weight (n = 37) had a maternal vitamin D level of 42.46 nmol/L (SD ±18.37), while full-term neonates with LBW (n = 27) had a maternal vitamin D level of 41.7 nmol/L (SD ± 22.17). Also, preterm neonates with normal birth weight (n = 30) had a maternal vitamin D level of 54.1 nmol/L (SD ± 27.81), while preterm neonates with LBW (n = 25) had a maternal vitamin D level of 41.28 nmol/L (SD ± 25.41) (Table 2).

**Table 2:** Mean Maternal Vitamin D Levels in Full-Term and Preterm Neonates

Mean Maternal Vitamin D	Normal Birth Weight	Low Birth Weight
Full-term neonates	42.46 nmol/L	41.7 nmol/L
Preterm neonates	54.1 nmol/L	41.28 nmol/L

## Discussion

In this study, maternal vitamin D levels were compared with the neonatal birth weight to establish a correlation between low maternal vitamin D level during pregnancy and LBW in neonates. However, the result showed no correlation between maternal vitamin D level and neonatal birth weight, which could be related to the fact that 87.4% of the mothers included in this study had low vitamin D (average vitamin D level = 44.97 nmol/L). Despite that, there was a difference in the mean maternal vitamin D levels, which were higher in mothers of neonates with normal birth weight (both full-term and preterm), as shown in Figure 1. Also, a study found that fetal birth weight raised 69 g per 1 ng/mL increase in maternal vitamin D and up to 20 ng/mL. Moreover, the decrease in maternal vitamin D level is associated with an increased risk of unadjusted small for gestational age (SGA) by 19% and adjusted SGA by 9% [10]. Multiple studies elaborated on the possible mechanisms of how maternal vitamin D level affects neonatal birth weight:

1) Low maternal vitamin D concentration may lead to suboptimal bone size and density after birth, which leads to LBW neonates [11].

2) Vitamin D has anti-inflammatory effect that regulates placental inflammation by inhibiting decidual NF-κB (nuclear factor kappa B) pathway, which is the main transcription factor of inflammatory mediators. Intrauterine growth restriction (IUGR) can be caused by maternal and placental inflammation, which shows that vitamin D deficiency can increase the risk of inflammation and therefore increase IUGR risk [9,12].

3) There is a relation between vitamin D and insulin-like growth factor (IGF-1) concentration, as vitamin D supplementation increases IGF-1 production in adults [9].

A mother who gets 600 IU daily supplement of vitamin D had increased level of vitamin D in cord and maternal blood and decreased IUGR [13]. Also, an increase in newborn’s length with the daily supplement was reported in a randomized controlled trial [14]. But using supplements from mid-pregnancy to six months postpartum did not improve fetal or neonatal growth [15].

Vitamin D has a major role in different fetal growth-related processes including fetal lung development, as it affects the maturation of alveolar type-2 cells and the epithelial-mesenchymal interactions. Furthermore, maternal vitamin D deficiency increased the risk of developing asthma in boys at the age of six years [16]. A daily dose of vitamin D supplement given to mothers in the second and third trimesters in a randomized controlled trial enhanced broad-spectrum proinflammatory cytokine response of cord blood mononuclear cells to innate and mitogenic stimuli. Also, it showed a 1.7- to 2.1-fold increase in levels of several proinflammatory cytokines (e.g. GM-CSF [granulocyte-macrophage colony-stimulating factor], IFN-γ [interferon gamma], interleukin [IL]-1β, IL-6, and IL-8) and a four-fold increase in IL-17A production. Therefore, maternal vitamin D is very important in the development of the fetal immune system, as an adequate level would protect the neonate from asthma-related outcomes including infections [17].

During infancy, vitamin D stores decline by 50% in less than a month, which causes rapid vitamin D deficiency if no supplements are provided [18]. Therefore, the American Academy of Pediatrics recommends that breastfed and partially breastfed infants be supplemented with 400 IU per day of vitamin D beginning in the first few days of life, as breast milk is not a sufficient source for vitamin D [19].

Vitamin D deficiency in children could cause multiple health issues, such as growth retardation, rickets, increased risk of certain cancers, hypertension, multiple sclerosis, and more severe forms of tuberculosis [20]. Also, growing children with low vitamin D are at a higher risk of developing type 1 diabetes mellitus [21].

## Conclusions

The result of this study showed no correlation between maternal vitamin D and neonatal birth weight, but mean maternal vitamin D levels were higher in mothers with normal birth weight neonates. Besides, 87.4% of mothers had low vitamin D levels during their pregnancy, with an average of 44.97 nmol/L. Therefore, further detailed studies on the Saudi population using the data of the primary health care centers across the kingdom are required to establish local guidelines about the treatment of vitamin D deficiency during pregnancy.
